# Protecting, managing and bending boundaries: a biomedicalization perspective on Swedish youth clinics’ responses to mental (ill) health

**DOI:** 10.1186/s12913-022-08259-w

**Published:** 2022-07-05

**Authors:** Isabel Goicolea, Maria Wiklund, Ida Linander, Linda Richter Sundberg

**Affiliations:** 1 Department of Epidemiology and Global Health, Umeå University, Umea, Sweden; 2 Unit of Physiotherapy, Department of Community Medicine and Rehabilitation, Umeå University, Umea, Sweden

**Keywords:** Mental health, Young people, Thematic analysis, Biomedicalization, Health care

## Abstract

**Background:**

Sweden has provided around 300 youth clinics (YCs) to address the health needs of young people since the 1970s. During the last few years, and as part of an effort to strengthen mental healthcare for young people, YCs’ role in the provision of mental healthcare has been widely debated. With such debates as background, the aim of this study is to analyse Swedish YCs’ responses to the mental (ill) healthcare needs of young people, from the perspective of national level stakeholders.

**Methods:**

We used thematic analysis of interviews with eight national level stakeholders in the field of youth mental health in Sweden. Building upon the concept of biomedicalization we examined the discourses on mental (ill) health, healthcare and youth that such responses reproduce.

**Results:**

YCs engage in the three simultaneous, but at times contradictory, responses of protecting, managing and bending boundaries. Remaining true to their mission as a health-promotion service compels them to protect their boundaries and limit the type of mental health issues they address. However, the perceived malfunctioning of specialized services has led them to bend these boundaries to allow in more young people with severe mental health problems. Caught between protecting and bending boundaries, the response of managing boundaries to decide who should be allowed in and who should be sent elsewhere has emerged as a middle-way response. However, it is not free from conflicts.

**Conclusion:**

Building upon the concept of biomedicalization, this study poses two questions. The first relates to whether it is possible to support young people and their health without reinforcing discourses that represent young people as collectively at risk, and if so how this can be done. The second relates to the provision of mental healthcare for young people, and the need to identify conditions for integrating diagnosis and treatment within YCs, without hindering their holistic and youth-centred approach.

## Introduction

In Sweden, youth clinics (YCs) have been responding to the health needs of young people for more than 40 years. YCs are well known in Sweden for their work on sexual and reproductive health, and they have been characterized from the beginning by a youth-centred approach [1]. This means that YCs address the diverse needs that each young user may have by means of a holistic approach to health, which includes aspects related to mental health and wellbeing [2]. However, it was not until 2016, when the Swedish government approved 130 million SEK to be given every year to YCs to strengthen their work on mental health, that they became visible nationwide as a key service for youth mental healthcare [3]. Hand in hand with the funding, discussions began about how to strengthen YCs’ response to mental health issues, and how this might affect the workings of YCs.

The increased interest in strengthening the work of YCs around mental healthcare has to be understood in relation to the time trends of increased reporting of psychiatric problems and utilization of psychiatric services, both in Sweden and globally [4, 5], as well as burgeoning media and political attention directed towards youth mental health [6, 7]. Alongside these developments, there is a perceived and reported inadequacy of existing mental health services to respond to the needs of children and young adults [6, 8]. From a broader perspective, these developments are taking place alongside a general restructuring of the Swedish welfare model [9, 10] towards more individualised neoliberal models. For example, when it comes to education, policy reforms in this direction have been linked with potentially negative effects on students’ health [11].

### First-line mental healthcare for young people

The Swedish public authorities’ response to these gaps in the mental healthcare for young people has been to launch the ‘First-line mental healthcare for children and youth policy’ (FLMHCY), with the aim ‘to provide the right help at the right time for young people who show early signs, or are at risk, of mental illness’ [6, 7]. This policy targets both the promotion of youth mental health and the prevention, diagnosis and treatment of mental ill health, conceptualized as mental (ill) health. The strategy of FLMHCY relies on involving more services to detect and address early signs of mental ill health among children and adolescents through, for example, engaging primary healthcare centres, school health services and YCs. The FLMHCY can be perceived as a shift towards community-based approaches that move away from biopsychiatry and its embracing of a medicalized model of psychiatric disorders [12].

It is important to highlight that FLMHCY services cannot function isolatedly but have to work in coordination with other services at a lower (community) and higher (specialized) levels in order to be able to offer comprehensive mental health care for young people. And that collaboration between such services that may hold different views and conceptualizations on mental (ill) health may not always be smooth [13].

The establishment of FLMHCY was expected to lead to positive health outcomes for children, youth and their families by providing early interventions and preventing more severe mental illnesses. In addition, it was anticipated that FLMHCY would relieve the high (unfulfilled) demand towards specialized child and youth psychiatry and strengthen the collaboration between diverse public services and sectors. The implementation of the policy has evolved, from assigning the first line mission to existing primary health care clinics and other services (e.g. youth clinics), to developing new services for triage and dealing with children and young people who do not require specialized care but need more than is available at the community level (e.g. school health) [6, 13]. The challenges of the mental health care system for young people are not unique to Sweden. Internationally, the literature highlights four key problems in the provision of mental healthcare for young people in most high-income countries: [1] poor penetration rate of services for the youth in need and a high rate of untreated prevalence; [2] delay in first contact and eventual treatment; [3] unsuitability of treatment for the particular stage of illness; and [4] serious problems with transitions between child and adult psychiatry [14]. Strengthening the role of first-line healthcare services targeting youth mental (ill) health [15, 16], as the Swedish FLMHCY policy aims to, is in fact part of a broader international approach that seeks to address this weakness.

Reforms of youth mental healthcare are ongoing in many high-income countries, but surprisingly there is still limited research analysing them [15, 17, 18]. Existing studies come mainly from the UK, Ireland, Canada and Australia, where the approach to FLMHCY relies on differentiated services for young people that are first-line and/or community based and address not only mental health issues, but also other youth healthcare (and sometimes social, educational and/or job-related) needs [19]. Such services are also referred to in the literature as ‘integrated community-based youth services hubs’ [20]. Research states that such models of integrating mental healthcare within first-line services have the potential to enhance the accessibility of mental healthcare for young people and to reduce delays in treatment [14, 15]. However, youth mental health programs implemented in primary services also face several barriers such as poor coordination between services, challenges in relation to transitioning between child, youth and adult services –and the fact that at least some of the young people accessing first line services experience more distress than what such services have been designed to address [14].

While research has been directed towards some of these models, like the Australian Headspace Programme [21, 22], the Jigsaw clinics in Ireland [23], and similar examples in the UK and Canada [20], to the best of our knowledge research analysing the Swedish case is lacking.

### Swedish youth clinics: an advantaged arena for integrating mental healthcare for young people?

Sweden has provided around 300 YCs, which have been working since the 1970s to respond to the healthcare needs of young people. According to a survey among young people using YCs in northern Sweden, YCs were rated highly in relation to several aspects of youth-friendliness such as accessibility, equity, respect, privacy and confidentiality, no judgement, and quality [24]. When it comes to offering first-line mental healthcare to young people, YCs have several advantages over other services – they are widespread, many employ professionals with expertise in mental (ill) health and, most importantly, they are trusted by young people [2]. The comprehensive approach of the YCs, in which professionals address the young user as a whole person and do not only focus on the primary reason for consultation, is also an important feature of YCs [1].

The relationship between YCs and the FLMHCY policy has been far from smooth. A mapping of mental healthcare available at Swedish YCs that was conducted in 2016 highlighted large variations in terms of staffing and opening hours, a lack of consensus about how mental healthcare work should be evaluated, documented and followed up, and huge heterogeneity in their responsibility towards mental healthcare: while some are responsible for working to promote good mental health and prevent mental health issues, others also have a responsibility to support and treat mental health conditions [25].

It is in this context of burgeoning political interest in strengthening youth mental (ill) healthcare through incorporating YCs into the FLMHCY, that we conducted this study. Our aim was to analyse Swedish YCs’ responses to the mental (ill) healthcare needs of young people, from the perspective of national-level stakeholders. We build upon the concept of biomedicalization to examine the discourses on mental (ill) health, healthcare and youth that such responses reproduce.

### Biomedicalization and healthcare systems’ responses to youth mental (ill) health

Clarke and colleagues used the concept of biomedicalization to capture a transformation in the organization and realm of medicine towards multi-sited processes of medicalization. Biomedicalization also illustrates a shift from control over medical phenomena to their transformation [26]. Three interlinked aspects of Clarke’s conceptualization of biomedicalization are especially relevant to this study: the expansion of the medical gaze to focus on health and life itself, the focus on risk prevention and an anticipatory orientation towards the future, and the internalization of lifestyle choices aimed at the continuous enhancement/optimization of health and life [26–28].

Biomedicalization proposes a view of the medical gaze as expanding beyond illness, disease and injury, to health and life itself. As Sweet expresses it, the shift from medicalization [29] to biomedicalization represents a shift from discipline to internalized control, from norms to optimizations […] and from a binary distinction between normal and abnormal to an ‘enhanced scale of normality’ (p.107) [30]. Inspired by the work of Foucault, the fact that, more and more frequently, care takes place outside the healthcare system is interpreted as an expansion of the clinical gaze [31] into people’s everyday lives, instead of a decrease in the power of the medical profession. This extension and diversification of the clinical gaze can be a way to interpret the focus of FLMHCY and other youth mental health policies on involving various less specialized services (such as primary healthcare or YCs), and less clinical spaces (like schools), in the early detection of young people at risk.

Biomedicalization also provides an alternative interpretation of the renewed focus on health and health promotion versus disease and curative services. This becomes especially relevant when analysing youth healthcare service models such as YCs or integrated, community-based youth service hubs. Instead of interpreting the focus of these models on promotion and prevention as a sign of decreasing medicalization (young users gaining control over their own health, less focus on diagnosing and treating diseases and more on promoting and sustaining health), the concept of biomedicalization analyses this turn in terms of health being represented as an individual goal, a social and moral responsibility, and a site for routine medical governance [26, 28]. As Lupton also points out (although she does not use the term biomedicalization), we are facing a ‘growing penetration of the clinical gaze into the everyday lives of citizens, including their emotional states, the nature of their interpersonal relationships, their management of “stress” and their “lifestyle” choices’ (p. 107) [32]. From a biomedicalization perspective, health becomes a commodity and, through the lifestyle choices that individuals should make, it is open to continuous enhancement [28]. As such, power and control over health is no longer only administered directly by the medical profession over patients/users, but also works through techniques of self-governance that patients/users internalize themselves [33, 34].

Clarke et al. [26] focus on the corporeality of enhancement and optimization processes (cosmetic surgery, anti-ageing, prosthetics, transplants), and the molecular and genetic levels (stem-cell research, prevention and treatment using molecular technologies). There are not many examples analysing optimization processes within the sphere of mental health (for noteworthy exceptions see [30, 35]). In this paper, we build upon Sweet’s criticism of biomedicalization analyses that ‘tend to emphasize high-tech interventions […] while most patients’ interactions with biomedicine proceed less fantastically’ (p.108) [30]. We argue that biomedicalization is also useful in the analysis of the ‘ordinary’ workings of youth mental healthcare services. Finally, despite the fact that a focus on the future is commonplace, both for biomedicalization and in some dominant discourses around youth, research on young people’s health is surprisingly absent from the biomedicalization literature. While youth as an ideal goal has been researched in relation to biomedicalization and ageing, to the best of our knowledge youth health and health care in itself has not received similar attention.

## Methodology

### Data collection

From November 2019 to September 2020, we conducted semi-structured interviews with a purposive sample of eight key stakeholders operating at the national level of the youth mental health system in Sweden. Four of the participants were representatives of the Swedish Association of Youth Clinics (FSUM). By default, FSUM representatives also work in YCs, providing services to young people or coordinating the work of YCs in specific regions. The other four stakeholders were representatives of national institutions or government agencies working on youth mental health policies.

The interviews were conducted in Swedish by LRS, face-to-face, via a digital meeting platform or by telephone. They lasted between 42 and 74 min. We explored topics of youth mental (ill) health, the Swedish youth mental health system and its current ability to respond to youth mental health care needs, the function and role of YCs, and possible areas of development within the system. The interviews were recorded and transcribed verbatim.

### Data analysis

We analysed the interviews following Braun and Clarke’s six phases for conducting thematic analysis [36][37]. The team involved in the analysis consisted of four qualitative researchers, two of them with both research and clinical experience working of youth mental health. Two of the researchers have worked with the concept of biomedicalization in other research projects.

We followed an emergent design, meaning that the analysis of the interviews guided our choice of concepts. In the first phase, we familiarized ourselves with the data, by reading the interviews several times individually and discussing preliminary impressions within the team. Afterwards, each of us read three or four interviews in more detail, coded them individually, and then discussed the codes within the research team. This guided the following phases of grouping the codes into candidate themes. It was during this phase that the team started discussing possible theoretical concepts and settled upon the concept of biomedicalization. In the fourth phase, IG and LRS checked the candidate themes in relation to the codes/data. Through writing and discussions within the group, we then refined the names given to the candidate themes. In addition, we started to explore linkages between the arguments we were making under each theme and the concept of biomedicalization. In the fifth phase, all team members provided written comments and again met for discussion. Building upon these discussions, IG redrafted the themes. In the final phase, the entire team revised this final draft and discussed, until we agreed on the final version of the results.

## Methodological discussion

Our study builds upon eight interviews, each generating rich data and multiple perspectives that, with the help of biomedicalization theory, allowed us to identify and expand upon dominant societal discourses on youth and mental health. Our participants were purposively selected to cover the most crucial institutions/organizations involved in these discussions. Since the data collection and analysis were conducted in parallel, we continued collecting data until we considered that the information gathered was enough to provide an answer to our research questions.

The concept of biomedicalization that we brought into the process during the analysis, following an emergent design, contributes to the conceptual transferability of our results. Biomedicalization allowed us to build upon the specific case of Swedish YCs in order to trace back dominant discourses on youth, healthcare and mental health. This makes our themes and arguments relevant beyond Swedish YCs.

### Findings

The three themes we developed summarize the three responses that participants argued YCs should provide in relation to mental (ill) health: ‘protecting boundaries – promoting healthy youth’, ‘managing boundaries – assessing the level of care’, and ‘bending boundaries – compensating for an insufficient specialized mental healthcare’.

### Protecting boundaries – promoting healthy youth

The ‘protecting boundaries’ response constitutes an endorsement of the idea that YCs should focus on mental health promotion, prevention and support – rather than treatment. The significance of focusing on the healthy aspects of youth mental health appeared repeatedly as a salutogenic discourse across the interviews. Linked to this definition of the response was also the perception that achieving it will be threatened if YCs start opening their boundaries to work more with mental ill health, as the FLMHCY assignment requires. Participant 1, from FSUM expressed these worries when asked about the challenges and opportunities of integrating the FLMHCY assignment within YCs:

*Yes, then the grounds for YCs disappear and what one imagines a YC is, that you should come here, you should work with self-empowerment, you should see the healthy, you should work with the whole, the whole person, the salutogenic approach, so to speak. […] Because now we’re going to start making diagnoses, too. Well, why? Is that really our job? Are we really going to categorize people in this way? Is it reasonable in adolescence, with the FLMHCY assignment, to do so or not? Do you understand? Then it becomes very much ‘sick-care’. And then it will bite us in the ass, or whatever you want to say, because then we’ll lose the trust of the young people.*

Participant 1 articulated a response that consists of promoting health, in contrast to providing ‘*sick-care’*. The Swedish word for healthcare can be directly translated as ‘sick-care’, so Participant 1 can be seen as stressing both that YCs should not focus on sickness, and also that YCs should be different from ordinary healthcare services. This discourse of ‘*strengthening the healthy*’ (as Participant 1 stated later during the same interview) lays at the core of YCs and distinguishes them from the rest of the healthcare system. The idea that YCs should focus on health promotion was unquestioned across the interviews, with the rationale behind this focus never being expressed explicitly. This prompts questions such as: what does it mean to ‘*strengthen the healthy’* young people? How is it that a part of the healthcare system is organized around healthy youth and not around illness?

A possible rationale for this focus on the healthy can be found in some references to youth being conceptualized as a*’special time’* that may require external support, especially in relation to sexuality and mental health, as Participant 2, from FSUM, described it:

*We agree that it’s a very special time in life, when a lot happens, physically and mentally. The brain grows so it cracks and the body grows in all directions. The hormones squirt. […] And then sexuality too… It’s existed all the time, of course, but in a different way… Since it’s precisely this combination, midwife and counsellor usually then [...] Thus, the sex and psyche connection. Because you don’t come here for a sore throat. It’s not healthcare in that sense.*

Hence, the focus on strengthening the healthy is perhaps connected to the construction of youth as a ‘*special time*’ (and maybe also a ‘healthy time’) and the response of YCs is understood as accompanying young people through this transitional phase to ensure that they reach adulthood safely. Or, as Participant 3, also from FSUM, put it: ‘*to help young people cross over to the adult world, and that they come out as strong individuals who can make good choices*’. Mental ill health then becomes a threat to this transition, and the promotional response of YCs consists of supporting young people to navigate youth and remain mentally healthy. Participant 1 further justified the importance of this response, not only for the young people themselves in the present, but also for the future:

*And what could be more important than working to make sure that our young people are well, get the help and support that’s needed? Because it’s they who will become the new citizens in society, who will take responsibility.*

Promoting healthy youth not only means that the primary target of YCs should be healthy young people, but also that YCs should approach the young people who come to the clinics *as healthy* – their worries and problems related to mental health should not be approached as psychiatric diagnoses but as variations along a continuum of what can be considered ‘normal’. Variations or instability in emotions and activity levels are interpreted as part of the transitional phase, not as symptoms of mental illness. Furthermore, this misinterpretation is considered potentially harmful to young people in that they will then identify with mental illness rather than embracing this variation in feelings and energy as normal. As Participant 4 described it in the next quotation, when asked about the main questions and consultations that young people bring to the YC:

*That I’m worried about the future, that I can’t sleep, that I’m feeling anxiety. That’s one of the most common reasons for young people to come ... if you want to talk about mental well-being, it’s anxiety, anxiety, stress. That’s what they want to talk about. Yes, and that’s part of life. Then it can lead to mental illness if you don’t get help dealing with it. And some have also got... suffer from mental illness, so to speak. But not everyone who feels like that is mentally ill, it’s normal.*

The above quote summarizes a topic that the participants repeatedly discussed: that the focus of YCs should be to approach certain feelings and emotions (such as sadness, worry, anger, nervousness) as normal and healthy aspects of growing up, without labelling them using psychiatric terms. But, while arguing that mental health diagnoses should be avoided, participants also argued that it is important not to overlook negative emotions, but rather to support young people to handle these emotions so that they do not progress to mental illness. It is important to detect early signs of potential risk that need to be handled in order to prevent problems in the future, as Participant 2 explained in relation to YCs:

*That you can come here as early as possible. I mean, the less trouble you have, the better. We’re thinking exactly the opposite of the rest of the healthcare system. I mean, ‘come as early as possible, so we can slow down [the deterioration in health]’. I mean, you don’t have to come here and say: ‘I think I have depression’. It’s enough to say ‘I’m feeling bad, this doesn’t feel good. I have some thoughts’. In other words, as early as possible so that we can work in as health-promoting a way as possible.*

The urge to normalize described under this theme gives professionals the power to decide what is normal and what is not, since it is the professional who ‘normalizes’ young people’s worries. This in turn contributes to the representation of mental health and mental ill health as separate entities, the possibility of setting boundaries between them, and the emergence of systems to establish these boundaries and distinctions. We expand upon this argument in the next theme.

### Managing boundaries – assessing the level of care

The ‘managing boundaries’ response refers to how YCs assess the level of care that young users will receive. As described in the previous theme, YCs’ main responsibility is towards mental health and mentally healthy young people, and participants argued that broadening their responsibility to address mental ill health would hinder their work. However, they also acknowledged that young people experiencing mental ill health do come to YCs. Young people do not think about whether or not the YC has a formal obligation to provide first-line mental healthcare, they just go there. Participant 2, from FSUM and also working in a YC at the time of the interview, reflected upon this when asked about the differences between YCs with and without the formal FLMHCY assignment: ‘*No matter what we call the YC, it’s the same people who come*’.

So, young people experiencing mental ill health do come to YCs and professionals there have to respond to them in some way. Participant 3, who had worked in YCs for many years and was involved with the FSUM directive at the time of the interviews, summarized what she considered the YCs’ response should be and what it should not be:

*We can’t make psychiatric assessments, we can’t. So when we talk about assessment, it’s just that we assess the [needed] level of care. And that’s something we call a social counsellor’s interview. […] So it’s just a matter of deciding ‘Are we the right place?’ And if we think we are the right place, we’ll initiate contact. […] So we say to young people: ‘we’re a low threshold service, but if we can’t help you, then we’ll help you to get to where you can find help.’*

Participant 3, together with other participants, argued that YCs should define, assess and guard the boundaries between what they can do in relation to mental ill health, and what they cannot do and will have to refer. Despite the triage and referral model seeming straightforward, the interviews also depict obstacles when attempting to embrace such a model. The first obstacle relates to the fact that such a system requires a well-functioning, specialized level to refer to, which, as we will describe in the next theme, is very seldom in place. The second obstacle refers to the fact that setting boundaries between what should be done at each level of the youth mental healthcare system was difficult. Participant 5, from a national governmental agency working with mental health, said:

*After all, there are still many ambiguities; what should be included in the first-line assignment, and where does the line between ... where is the sharp boundary between the first-line and specialized psychiatry, when to refer patients, this is also unclear. The first-line assignment also looks different in different regions. Who has the assignment also looks very different.*

Hence, difficulties in determining which mental healthcare services could be offered by YCs, and which should be referred to other services, needs to be contextualized within the existing FLMHCY policy. Participants also argued that this policy does not make these boundaries clear enough, and the heterogeneity in how the FLMHCY assignment has been adopted in the different regions makes boundaries even more blurred and unclear.

Participant 6, a civil servant who was directly involved in the discussions around the implementation of FLMHCY, reflected upon the tensions in relation to YCs’ role in mental ill health, and what she considered to be the conundrum of the debate:

In one it said mental illness, in the other it said mental health, and that led to a huge discussion. Have we thought that the YCs should treat mental illness or should they just promote mental health?

Participant 6 left this question unanswered. However, the fact that such a question is even asked brings with it certain assumptions and opens up certain possibilities: if YCs have to decide whether to treat mental illness or to promote mental healthcare, this means that the two are different and distinguishable phenomena, and that each of them can be addressed differently and in different arenas.

### Bending boundaries – compensating for insufficient specialized mental healthcare

‘Bending boundaries’ refers to the situation in which YCs respond to (severe) mental health problems as a way to compensate for the inadequacy of specialized services. The two previous themes describe the need for YCs to distinguish between who they should and can care for, and who should be referred elsewhere, and how setting such boundaries is required to safeguard the role of YCs in promoting healthy youth. The message was clear: YCs should not overstretch their boundaries to treat mentally ill young people. However, at the same time, the interviews captured many experiences of YCs and their professionals ‘bending these boundaries’ to compensate for shortages in the wider mental healthcare system, as Participant 4 explained:

*But unfortunately, now, in many places the YC has also become the place that has to take care of those who feel really bad, who should perhaps be with child and adolescent psychiatry, or in adult psychiatry. They go... they’re in YCs and are held there for too long as well, in some ways. And then there’s no time for these other things* [that YCs should be working with].

The descriptions of the inaccessibility of specialized mental health services and the consequences for young people – and YCs – were robust in the material. Maintaining strict boundaries created frustration among YCs’ staff because it prevented them from offering more to individual young people. The response of bending boundaries thus emerges as the YCs navigate this fact in order to ensure that the young people get access to the right care.

However, bending boundaries and doing more in relation to mental ill health also harmed YCs’ staff. Participant 3, for example, described how, in an effort to compensate for the shortcomings of specialized psychiatric services, YCs staff started dealing with ‘*heavy things, which we did not have the supervision and competence to deal with. And then, as a consequence, there has been sick leave at the YC.*’

Without extra resources, organizational changes, the necessary competences, backup staff and a formal assignment, bending boundaries led to YCs experiencing longer waiting times and staff burnout. This was an argument against such a strategy. As Participant 4 summarized:

*If there was a national assignment with resources for [YCs]… then I think it would be a perfect place […] to have a first-line assignment at a YC. But then you also need the staff categories that are able to make an assessment. But I think like here, psychiatrists, psychologists, counsellors, and so on. In team work […] Then I think it would be perfect. But as it is now, with it… then it’s difficult. So, I know… in some places, the YC has been given a formal assignment in the healthcare region. And it works well, as far as I know, relatively well in some places and in some places it will instead be that there will be very long queues. Most often, perhaps, the other thing that is the YCs’ work may be squeezed out.*

While YCs could possess good prerequisites for taking on a more active role in responding to mental ill health, there are also certain aspects lacking in terms of available resources. Bending boundaries under such circumstances comes with harmful effects for both the young users and the staff of YCs, as well as for the overall mission and YCs’ way of working. Under the current circumstances, bending boundaries may mean that YCs, as we know them, may cease to exist.

## Discussion

In responding to young people’s mental healthcare needs and national policies, YCs engage in the three simultaneous, but at times contradictory, responses of protecting, managing and bending boundaries. Remaining true to their core mission as a low-threshold health-promotion service compels them to protect their boundaries and limit the type of mental health issues that, according to the FLMHCY, they should address. However, the perceived malfunctioning of specialized services and YCs’ commitment towards youth leads them to, sometimes bend these boundaries to allow in more young people with more severe mental health problems. Caught in between what they perceive as their core mission (promote health, avoid making psychiatric diagnoses) and the fact that young people with different mental health problems do present at YCs, managing boundaries to decide who should be allowed in and who should go somewhere else emerges as a middle-way response, which is not free from conflicts.

Using the concept of biomedicalization to theorize our results [26–28], this study raises two crucial questions that future mental health programmes and policies for youth should bear in mind. The first relates to whether it is possible to support young people and their mental health without reinforcing discourses that represent young people as particularly vulnerable and collectively at risk [12], and if so how this could be achieved. The second relates to the provision of mental healthcare for young people, and the need to identify conditions for integrating diagnosis and treatment within community-based youth mental health services, without hindering their holistic and youth-centred approach. Alternatively (or in conjunction), there is a need to identify conditions to ensure that services to diagnose and treat young people with mental ill health can offer the same holistic and youth-centred approach as community-based services, such as YCs. There are models, like Open Dialogue, that aim to strengthen continuity by strengthening collaboration and relying on teams of professionals from various levels to provide care that is centred on the specific needs of each individual user [38, 39]. However, Open Dialogue has focused on addressing the needs of users who had psychiatric diagnosis. To what extent and how such model could include young people who may not require (or fulfil the criteria to get) psychiatric diagnosis has, to the extent of our knowledge, not been studied.

### Youth and their mental health as the object of the healthcare system

YCs’ reluctance to engage with mental ill health diagnoses and treatment is congruent with their focus on making ‘normal’ youth their target. This focus could be interpreted as a form of resistance to biopsychiatry and its embracing of a medicalized model of psychiatric disorders [12]. This approach of avoiding pathologizing mental health symptoms and diagnosis and instead promote personal autonomy and social network involvement is also in line with contemporary approaches of organizing and conceptualizing mental health services, seen in for example the Open-dialogue approach [38, 39]. In our study this is demonstrated, for example, in YCs’ hesitancy to use medical-psychiatric terms despite the fact that young people themselves may utilize such language. An approach that considers sadness, grief, anxiety or stress as normal can result in the destigmatizing of certain experiences of mental ill health and a broadening of the spectrum of what it may be considered ‘normal’ to experience without being considered sickness. On the other hand, it can also risk trivializing young people’s self-perceived health problems, worries and concerns, since they are deemed ‘normal’.

Our results align with how the Swedish Association of YCs describes the core mission of these services: to focus on youth mental health instead of on sickness, prioritizing prevention and promotion, and reaching every young person by making healthcare services available nearby [1]. Such an approach also forms the basis for the community-based mental health models that are currently being implemented in several countries worldwide [15, 20]. Such approaches aim to improve access and reduce system fragmentation, while providing a single point of entry to comprehensive, evidence-based services [20]. Efforts to develop policies to strengthen such approaches speak of the responsibility of the healthcare system towards youth, which can be interpreted as increased attention being directed towards young people and their needs.

From a biomedicalization approach, however, we can also interpret the focus on ‘normal’ youth and targeting healthy young people as an expansion of the mission of the health system to address life itself [28, 40] for one specific group: young people, who thus become the target of the health system. The expansion of the medical gaze into health and life itself means that young people no longer need to have particular symptoms to be considered at risk; instead, they are all constructed as being in the potential process of becoming ill [41] and, hence, legitimate subjects of health-related discourses [26]. Such a focus contributes to representing young people as a group for whom risks are seen to be higher [35, 40]. Thus, they require the knowledge and support of expert professionals in order to be properly monitored, reassured and corrected in relation to their own diagnosis, and supported in making an appropriate transition towards adulthood.

From a biomedicalization perspective, the role of medicine then becomes not only to monitor, reduce and manage risks but also to reshape the way in which we understand our bodies and lives as always open for enhancement. The aim of empowering and strengthening young people in this way can be connected with technologies of the self, forms of self-governance that people internalize [26, 34, 41]. In this case, enhancement to make the best of oneself becomes the individual responsibility of every young person [28, 40, 42]. The regulation of young people is no longer only achieved through the direct intervention of medical professionals, but also through behavioural and lifestyle modifications internalized by young people as they seek a transformation of their health and, ultimately, themselves [26, 43].

### Creating categories, assessing and sorting youth and their needs

Our results also highlight that healthcare services focusing on enhancing the normal and the healthy cannot exist without establishing boundaries. The problem with drawing boundaries around first-line youth mental healthcare services is not unique to Sweden. Similar models of integrated community-based youth service hubs face the problem of how to respond to young people who present with complex mental healthcare needs to services that were initially designed with promotion and prevention in focus [14, 19, 20]. To note, as users of mental health services young people can be understood as ‘active agents’ or ‘consumers’ [44, 45], also with the right to be engaged and involved in decision-making [46]. The involvement of youth in their healing is a key feature in the recovery literature that implies a shift of perspective from the health provider to the individual and personal perspective on the experience of mental health and the recovery from mental health problems [47, 48]. Digital health technologies and social media are relatively new arenas where they can seek and produce knowledge, and share experiences, to make sense of their bodies and health [44]. By this, also youth themselves are engaged in creating categories, assessing and sorting their needs.

A mental health subsystem that is structured into different levels of care requires that young people are sorted, in a process of ‘triage’, into categories and cared for in different places. Triaging to distinguish between who can be cared for within YCs, and who cannot, not only organizes the work and divides responsibilities but also contributes to representing two distinct youth subpopulations: those who are mentally healthy, and those who are mentally sick, and creates a division between mental health and mental sickness. Such a division can be interpreted as building upon biopsychiatric conceptualizations of mental illness and health as a single bipolar dimension, with mental health at one of the extremes and mental illness at the other [49]. Within such a binary conceptualization, YCs should only deal with mental health, and specialized psychiatric services should deal with sickness/diagnosis. On the other hand, YCs’ focus on ‘the normal’ and ‘the healthy’ can also be interpreted as a conceptualization similar to Keyes’ continuum of mental health, which considers that, instead of being opposite extremes, mental health and illness are ‘distinct but correlated axes’ (p. 546)[49]. Under such a conceptualization, mental health should be addressed in its own right, being itself a continuum between flourishing and languishing – where languishing does not equate with mental illness. Our results highlight that both conceptualizations (binary, continuum) seem to coexist within the Swedish mental healthcare subsystem. In this scenario, it becomes difficult for YCs to reconcile a focus on mental health in its own right (aiming to promote the flourishing of mental health) while at the same time approaching it as one extreme of a binary (aiming to prevent progression to mental ill health). We agree with Sweet [30] that, while continuum discourses may suggest that ‘we have moved away from binaristic notions of normality and wellness, binaries still operate importantly in psychiatric discourses, even those that circulate as progressive and humanistic’ (p. 105). To summarize, while YCs focus on ‘the normal’ and enhancement can be interpreted as a shift towards a conceptualization of normality as a continuum, such a continuum does not encompass every experience of mental ill health.

The process of setting boundaries for what constitutes mental illness is often based on expert knowledge [26]. From our results, the complaints articulated by young people who present at YCs, for example anxiety, were deemed by the participants to differ from mental ill health. This shows that anxiety in its diagnostic form is understood as something for healthcare professionals to judge and decide upon, not the young people themselves. This can be connected to the idea of expert positions and knowledge having the power and means to distinguish between the mentally healthy and the mentally ill [27, 28]. The creation of boundaries and classifications requires an apparatus for its own functioning and preservation: expert professionals who are able to judge, instruments to make such classifications, and referral pathways to link one service with another, to name just a few. On the one hand, such a system creates possibilities, in terms of access to wider or specialized resources, the use of standards that are less arbitrary, and facilitate monitoring and evaluation, which can enhance quality. On the other hand, the complexity of the system and the fact that it reproduces the mainstream way of organizing healthcare makes it difficult to challenge and think of other, alternative organizational forms. The possibility of having services that could deal with the whole spectrum of mental health and ill health was, in fact, never mentioned. This could be tracked back to how mental healthcare, in Sweden and elsewhere, is organized around diagnosis and/or stages. In this way, the mind/body divide is also reproduced, and mental and physical/somatic health are kept separated – all according to a biomedical model of organizing health services.

Establishing diagnostic categories and separating the healthy from the sick, the body from the mind, lies at the core of medicalization processes [29], of which biopsychiatry is an exponent [12]. Such a structure allows for increasing specialization, which, arguably, can provide a better response to needs that require specific competences. However, specialization has also been criticized for contributing to fragmentation, and as not being the best way to approach bio-psycho-social complexity [50], which is often the situation in youth mental health. While the benefit of this specialized way of organizing is that it claims to offer more adequate care due to different needs being catered for in different spaces that are appropriately specialized, our results indicate that such boundary creation makes it harder for young users to navigate the system and to find the care they need. If holistic and youth-centred care is equated with prevention, promotion and health and the work of first-line services, then those young people who are considered too mentally unwell might be left out of prevention and promotion initiatives. Here, while the low threshold of YCs and similar services is imagined to improve access for young people, we can also reimagine it as limiting this access to certain groups of young people, those who, in fact, may be most in need of care and support.

## Conclusion

Our findings draw attention to some important points that youth mental health subsystems should consider. Firstly, the need to bring young people and their health onto the political agenda in a way that does not contribute to reproducing discourses of young people as an at risk group [35, 40] and youth as deficient, while at the same time not idealizing youth as a period free from problems and challenges or trivializing young people’s needs. Secondly, the need to strengthen mental healthcare in a way that facilitates continuity of care for young people and ensures that young people experiencing mental ill health can also receive support for other health issues beyond their mental health diagnosis.

In sum, a focus on ‘normal youth’ and on mental health promotion can contribute to reproducing a deficit discourse on youth and may reinforce the creation of categories (and hierarchies) and stratified care [28]: some young people may easily gain access to comprehensive care that includes promotional activities, sexual and reproductive health information and care, while others will receive care that only focuses on their mental health diagnoses, while neglecting their other needs. However, pointing out the problematic possibilities of biomedicalization processes is only part of the picture. Our findings also highlight how, within a shrinking welfare state, services like YCs play a key role not only in responding to the diverse needs of young people within a context where other services have ceased to play that role, but also in advocating for young people and their mental health as a crucial societal responsibility.

## Data Availability

The dataset analysed during the current study is not publicly available because it contains sensitive information, but it is available from the corresponding author on reasonable request.

## Abbreviations

YC: Youth clinic
FLMHCY: First-line mental healthcare for children and youth policy
